# Exploiting changes in the tumour microenvironment with sequential cytokine and matrix metalloprotease inhibitor treatment in a murine breast cancer model

**DOI:** 10.1054/bjoc.2000.1487

**Published:** 2000-11-22

**Authors:** K A Scott, H Holdsworth, F R Balkwill, S Dias

**Affiliations:** Biological Therapies Laboratory, Imperial Cancer Research Fund, 44 Lincoln's Inn Fields, London, WC2A 3PX, UK

**Keywords:** IL-12, MMP-9, TIMP-1, BB94, MMPI, mammary carcinoma

## Abstract

The study of treatment-induced changes in the tumour microenvironment might lead to effective combinations of biological therapy. IL-12 induced tumour regression and cure of an experimental murine breast cancer, HTH-K, but only after long-term treatment that was associated with chronic toxicity. During IL-12 therapy, tumour levels of the matrix metalloprotease MMP-9 declined and its inhibitor TIMP-1 was strongly induced. We therefore administered alternate cycles of IL-12 and the MMP inhibitor Batimastat (BB94) to mice. Therapeutic efficacy was increased compared with short-term IL-12 therapy but without the chronic toxicity associated with long-term IL-12 treatment. Image analysis of treated tumours revealed that BB94 prevented regeneration of tumour and stromal compartments that normally occurred after short-term IL-12 therapy. © 2000 Cancer Research Campaign http://www.bjcancer.com


*British Journal of Cancer* (2000) **83**(11), 1538–1543
© 2000 Cancer Research Campaign
doi: 10.1054/ bjoc.2000.1487, available online at http://www.idealibrary.com on IDEAL®

http://www.bjcancer.com

### Exploiting changes in the tumour microenvironment with sequential cytokine and matrix metalloprotease inhibitor treatment in a murine breast cancer model

**KA Scott, H Holdsworth, FR Balkwill and S Dias**

Biological Therapies Laboratory, Imperial Cancer Research Fund, 44 Lincoln's Inn Fields, London WC2A 3PX, UK

Summary The study of treatment-induced changes in the tumour microenvironment might lead to effective combinations of biological therapy. IL-12 induced tumour regression and cure of an experimental murine breast cancer, HTH-K, but only after long-term treatment that was associated with chronic toxicity. During IL-12 therapy, tumour levels of the matrix metalloprotease MMP-9 declined and its inhibitor TIMP-1 was strongly induced. We therefore administered alternate cycles of IL-12 and the MMP inhibitor Batimastat (BB94) to mice. Therapeutic efficacy was increased compared with short-term IL-12 therapy but without the chronic toxicity associated with long-term IL-12 treatment. Image analysis of treated tumours revealed that BB94 prevented regeneration of tumour and stromal compartments that normally occurred after short-term IL-12 therapy. © 2000 Cancer Research Campaign http://www.bjcancer.com

**Keywords:** IL-12; MMP-9; TIMP-1; BB94; MMPI; mammary carcinoma

By analogy with successful chemotherapy regimes, it is likely that biological therapies such as cytokines, angiostatic agents and matrix metalloprotease inhibitors, will work best in combination. However, this may increase toxicity without increasing efficacy. Sequential use of biological therapies directed against different tumour and stromal interactions may be effective and less toxic, but guidance is needed in determining appropriate agents and schedules. A better understanding of the effects of biological therapies in the tumour microenvironment may lead to rational design of combination biological therapy.

The cytokine interleukin-12, IL-12, has anti-tumour actions in a range of experimental models but in these, and in clinical trials, it has undesirable side effects. The signs of IL-12 toxicity include splenomegaly, due to extramedullary haematopoiesis, hepatic necrosis, lethargy and weight loss (Tare et al, 1995; Myers et al, 1998). Preclinical animal studies of IL-12 action are notable for their detailed study of the tumour microenvironment (Tannenbaum et al, 1996; Tsung et al, 1997; Cavallo et al, 1999), where a number of distinct events precede tumour regression, many of which are attributed to the local induction of IFN-γ (Nastala et al, 1994; Dias et al, 1998a; Ogawa et al, 1998).

IL-12 induces tumour regression in mice bearing the experimental murine breast cancer HTH-K (Dias et al, 1998a, 1998b) with a distinct sequence of events including induction of IFN-γ-inducible genes; an influx of cytotoxic T cells; apoptosis of tumour cells and destruction/inhibition of tumour blood vessels. The anti-angiogenic action of IL-12 in the HTH-K model involved at least 2 mechanisms; decreased VEGF production by the tumour cells and reduction of MMP-9 levels at the tumour site (Dias et al, 1998b). The reduction in MMP-9 was accompanied by an increase in TIMP-1, the natural inhibitor of MMP-9. However, prolonged IL-12 therapy, for 30 days or more, was necessary for sustained regression and cure of mice bearing HTH-K tumour, and this was significantly toxic (Dias et al, 1998a).

As we observed a change in the MMP/TIMP balance in the tumour microenvironment after 14 days of IL-12 therapy, we reasoned that a synthetic MMP inhibitor might enhance or consolidate the effects of IL-12 without undue toxicity. In this paper we report that alternating cycles of IL-12 and the MMP inhibitor Batimastat (BB94) increased therapeutic efficacy without increasing toxicity. Administration of BB94 appeared to prevent regeneration of tumour and stromal compartments that normally occurs after short-term IL-12 treatment.

*Received 20 March 2000*
*Revised 12 July 2000*
*Accepted 18 July 2000*

*Correspondence to:* FR Balkwill

### MATERIALS AND METHODS

### Mice

Female BALB/c mice from the Specific Pathogen Free (SPF) Unit [Clare Hall Laboratories, Imperial Cancer Research Fund (ICRF), South Mimms, UK], 5–6 weeks of age, were used in all experiments. All animal work was carried out according to the guidelines specified by the UK Home Office Animals Scientific Procedures Act 1986.

### IL-12 and BB94

IL-12 was a gift of Stan Wolf from Genetics Institute (Cambridge, MA) and had a specific activity of $3.3 \times 10^6$ U mg$^{-1}$. IL-12 was diluted to 10 μg $ml^{-1}$ in PBS/0.1% murine serum albumin (Sigma Chemicals, UK) and stored at –70˚C prior to use. Mice were treated with 1 μg IL-12 $day^{-1}$, as previously described (Dias et al, 1998a). BB94 was kindly provided by British Biotech Pharmaceuticals (Oxford, UK). It was diluted in 1 in 8 with dextrose for injection, and used at a final concentration of 40 mg $kg^{-1}$ $day^{-1}$.

### Tumour implantation

The HTH-K tumour model (a syngeneic breast carcinoma) was transplanted subcutaneously from mouse to mouse. All treatments were given i.p. and started 3 days after tumour implantation. Control groups were treated with PBS containing 0.1% murine serum albumin or BB94 diluent control (dextrose solution). The dimensions of the subcutaneous tumours were recorded twice weekly and mice were killed when the tumour reached a volume of 1.2 $cm^3$. Tumour volume was calculated according to the formula $A^2 \times B/2$ where A is length and B is width.

### Protein extraction

Soluble protein was isolated from 100 mg of tissue using 2 ml of ice-cold 1.5% (w/v) Triton-X-114 in PBS (Sigma). The tumour sample was homogenized in 1.5% (w/v) Triton-X-114 using an Ultra-Turrax T25 homogenizer until fully disrupted and then incubated on a roller at 4˚C overnight. Any undigested tissue was removed by centrifugation at 13 000 ***g*** for 10 min. The supernatant was subsequently incubated at 37˚C for 5 min followed by centrifuging at 13 000 ***g*** for 5 min. The upper aqueous phase containing soluble proteins was removed and total protein concentration was determined using the Biorad protein assay kit (Biorad, Hemel Hempstead, UK) according to manufacturer's instructions.

### Type IV collagenolytic assay

The ability of samples to degrade type IV collagen was determined using $^3$H radiolabelled type IV collagen (NEN, Boston, USA) using the protocol described previously with minor modifications (Fisher and Werb, 1995). Briefly $^3$H type IV collagen (NEN) was dissolved in assay buffer (50 mM Tris-HCl pH 7.5 containing 0.2 M NaCl and 10 mM $CaCl_2$) such that each tube contained 5 000–10 000 cpm. Samples were added and the tubes were incubated at 37˚C overnight. To precipitate undigested $^3$H collagen, 10% (w/v) TCA containing 0.5% (w/v) tannic acid was added. Samples were incubated on ice for 30 min followed by centrifugation at 5000 ***g*** for 15 min. Type IV collagenolytic activity was measured by scintillation counting of the supernatant. A positive control of 100 U $ml^{-1}$ bacterial collagenase (Sigma) and a negative control of 20 mM EDTA were employed. Collagenolytic activity was measured in duplicate from 2 mice in each group at every time point.

### Histology and quantitative microscopy

At postmortem examination, samples were removed and fixed in formal saline, embedded in paraffin and sectioned by the ICRF Histopathology Unit according to standard procedures. Histology was evaluated in haemotoxylin & eosin stained sections. The percentage area occupied by tumour cells, stroma, blood vessels and necrosis in tumours treated with either IL-12, BB94 or both were analysed at various time points during tumour growth. Sections were observed using a Nikon Labophot II microscope (Nikon, Kingston, UK) at a magnification of ×100 (10 × objective and 10× eyepiece) and analysed using Aequitas 1A image analysis software (Dynamic Data Links, Cambridge, UK). Complete sections were analysed from at least 2 mice (2 sections per mouse) from each group at a given time point. Mean percentage area (±SEM) occupied by tumour cells, stroma, blood vessels and necrosis was calculated from low power observation (×100) of the whole section. This was between 18–50 fields of view dependent on number of mice and size of tumour.

### Statistical analysis

Statistical evaluation of survival data was estimated using either the Cox proportional hazards model (Figure 3A and 3B) with tied values handled by evaluating the log-likelihood function by the exact partial method, or the stratified logrank test (Figure 2A). Differences in tumour volumes according to treatment administered were investigated at each measurement time by analysis of variance (ANOVA). All statistical evaluations were performed by Mike Bradburn at the ICRF Medical Statistics Group (Institute for Health Sciences, Oxford, UK).

### RESULTS

### Treatment with IL-12 reduces MMP-9 expression and activity

Using the technique of gelatinolytic zymography on tumour lysates, we previously demonstrated that IL-12 inhibited MMP-9 production in the tumour microenvironment (Dias et al, 1998b). However, during zymography, MMPs are dissociated from their natural inhibitors. We therefore assessed the net collagenolytic activity in the tumour microenvironment by the ability of tumour lysates to degrade radiolabelled type IV collagen. As shown in Figure 1, collagenolytic activity increased steadily in control mice. At day 5 collagenolytic activity was 4-fold higher in tumours treated with IL-12 compared with placebo. This was probably due

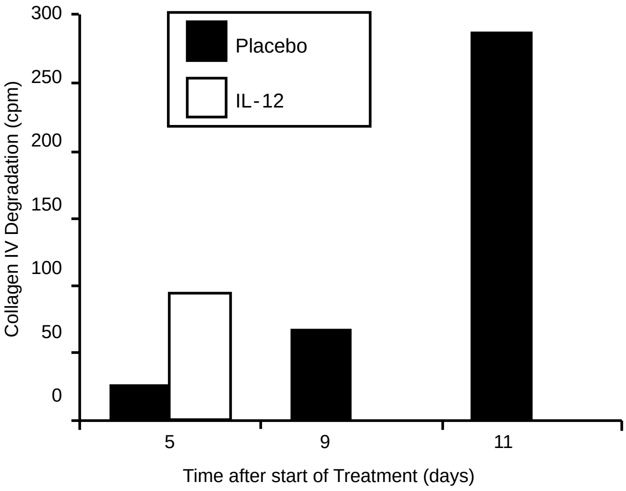

**Figure 1** Type IV collagenolytic activity was measured in placebo and IL-12 treated HTH-K tumours by degradation of $^3$H type IV collagen. Six mice were treated with IL-12 or placebo and 2 mice per treatment were culled at each time point. The results are representative of 2 experiments. Results were expressed as mean radioactivity (cpm) released into the supernatant

© 2000 Cancer Research Campaign to MMP-9 production by the macrophage infiltrate which occurs following IL-12 treatment (Dias et al, 1998a). By day 9 collagenolytic activity was not detectable in IL-12 treated tumours but had increased 2-fold in placebo mice. At day 11 the level of collagenolytic activity had increased to more than 10-fold the activity in placebo mice at day 5. Once again there was no collagenolytic activity in IL-12 treated mice at day 11.

### IL-12 prolongs survival and reduces tumour volume of HTH-K bearing mice

We previously showed that the efficacy of IL-12 was related to length of treatment. 12 or 15 days therapy increased the survival of HTH-K bearing mice 2- and 5-fold, but complete tumour regression was only achieved in 1/8 mice treated for 15 days compared with 7/8 mice treated for 45 days (Dias et al, 1998a).

Figure 2A shows that the survival of HTH-K bearing mice treated with IL-12 for 10 days was 2-fold greater than control mice ($P < 0.0001$). HTH-K grew at a rapid rate in placebo mice reaching a maximum volume of 1.2 $cm^3$ at 10 days (Figure 2B). Tumour growth was delayed in mice treated with IL-12 ($P < 0.0001$ compared with placebo and BB94-treated groups). There was a lag period of 15 days before any major increase in tumour volume occurred (Figure 2B).

### Sequential treatment with IL-12 and BB94 enhances the effect of IL-12

BB94 was administered for 10 days alone or after 10 days of IL-12 treatment. As a single agent, BB94 had little effect on survival of tumour-bearing mice (Figure 2A), although a slight growth delay and a reduction in tumour volume was noted (Figure 2B). Sequential treatment with IL-12 (10 days) followed by BB94 (10 days) significantly increased mouse survival when compared with IL-12 treatment alone ($P = 0.05$). Another treatment schedule of IL-12 (10 days), BB94 (10 days) followed by IL-12 (10 days) resulted in a 3.5-fold increase in survival compared to IL-12 treatment alone (data not shown, $P = 0.002$). At 19 days tumour volume was significantly smaller in IL-12/BB94 ($P$=0.01) and IL-12/BB94/IL-12 ($P < 0.001$) (data not shown) treated mice as compared with IL-12 treatment alone. This sequential (IL-12/BB94/IL-12) treatment resulted in 1/6 complete tumour regressions. Similar results were seen in a second experiment with the same schedule.

### Optimization of the treatment schedule induces complete tumour regression

The treatment cycle for each agent was then extended from 10 to 14 days. Treatment of mice with IL-12 for 14 days resulted in a 5-fold increase in survival compared with placebo ($P < 0.0001$) (Figure 3A). Once again administration of BB94 alone had little effect on survival. Tumour-bearing mice treated with IL-12 (14 days) followed by BB94 (14 days) survived slightly longer than mice treated with IL-12 alone with 1/12 mice obtaining complete tumour regression (Figure 3B). 29 days after the start of therapy 1/12 (IL-12), 4/12 (IL-12/BB94) and 6/12 (IL-12/BB94/IL-12) mice were still alive (significant difference between IL-12 treated and sequential treatments at 29 days $P = 0.04$). It is important to note that had a group been included with the schedule IL-12 (14 days), placebo (14 days) and another dose of IL-12 for 14 days there would have only been one mouse to treat by the time the second IL-12 cycle was due.

Thus the influential time for survival appears to be between 10 and 15 days after the first cycle of IL-12 was completed. BB94 enables some mice to survive through this period by preventing tumour regrowth.

The most potent schedule was IL-12/BB94/IL-12 (in 14 day cycles). Survival of mice was significantly prolonged compared with mice treated with 14 daily administrations of IL-12 ($P = 0.002$) and IL-12/BB94 treated mice ($P = 0.03$) (Figure 3B). At 38 days 6/12 mice were still alive in this treatment group compared with 0/12 mice treated with IL-12 alone. 4 mice (out of the 6/12 still alive at 38 days) achieved complete tumour regression and had no recurrence of disease after 300 days.

### Sequential treatment of IL-12 and BB94 results in angiogenesis blockade

Examination of H&E stained tumour sections from mice receiving the different treatment schedules suggested that BB94 might be delaying the regeneration of the tumour tissue that generally

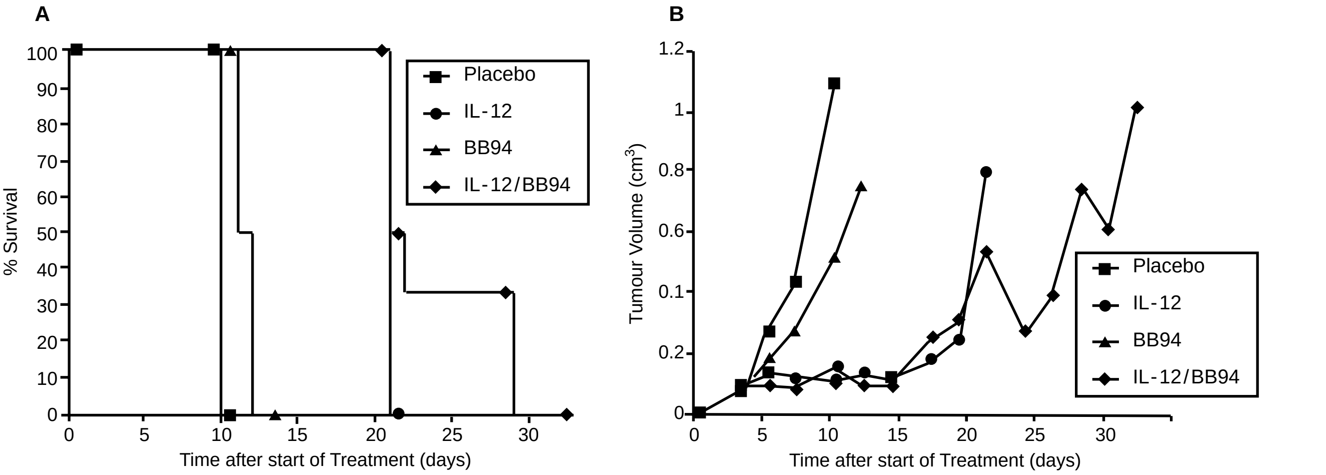

**Figure 2** Survival and tumour volume of HTH-K bearing mice. Six mice per group were treated with one of the following schedules: placebo, IL-12 10 days, BB94 10 days, IL-12 10 days followed by BB94 10 days. Percentage survival of HTH-K bearing mice (**A**). HTH-K tumour volume measured as described in materials and methods (**B**)

*© 2000 Cancer Research Campaign*

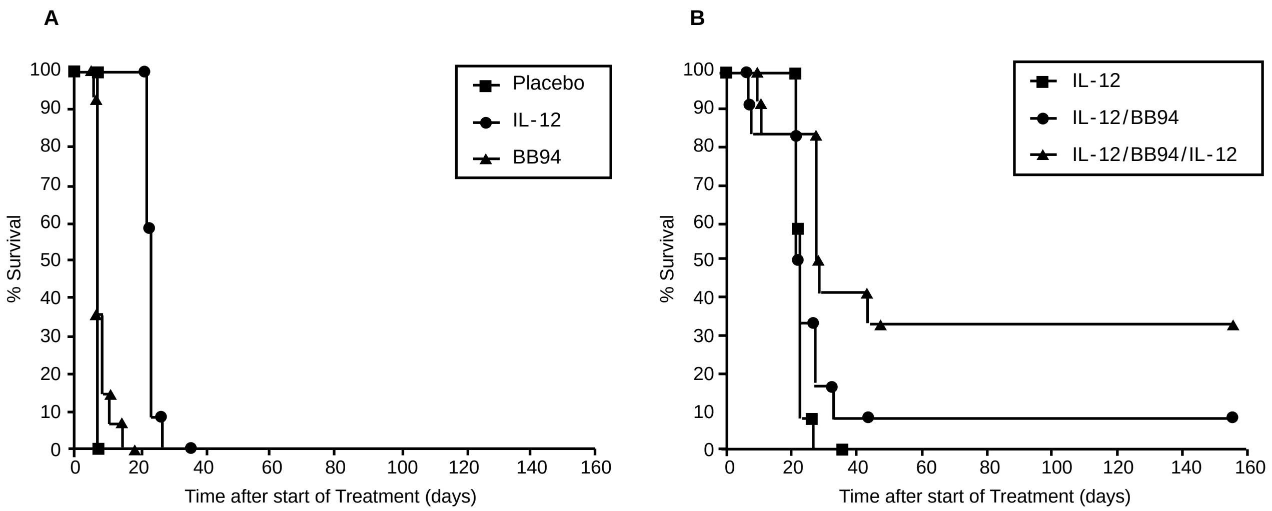

**Figure 3** Percentage survival of HTH-K tumour bearing mice following optimization of sequential therapy. Treatment schedules were extended to 14 days; placebo, IL-12 14 days, IL-12 14 days followed by BB94 14 days, IL-12 14 days followed by BB94 14 days followed by IL-12 14 days. Each group contained 12 mice. Percentage survival of HTH-K bearing mice treated with a single agent (**A**). Percentage survival of HTH-K bearing mice with sequential treatments (**B**)

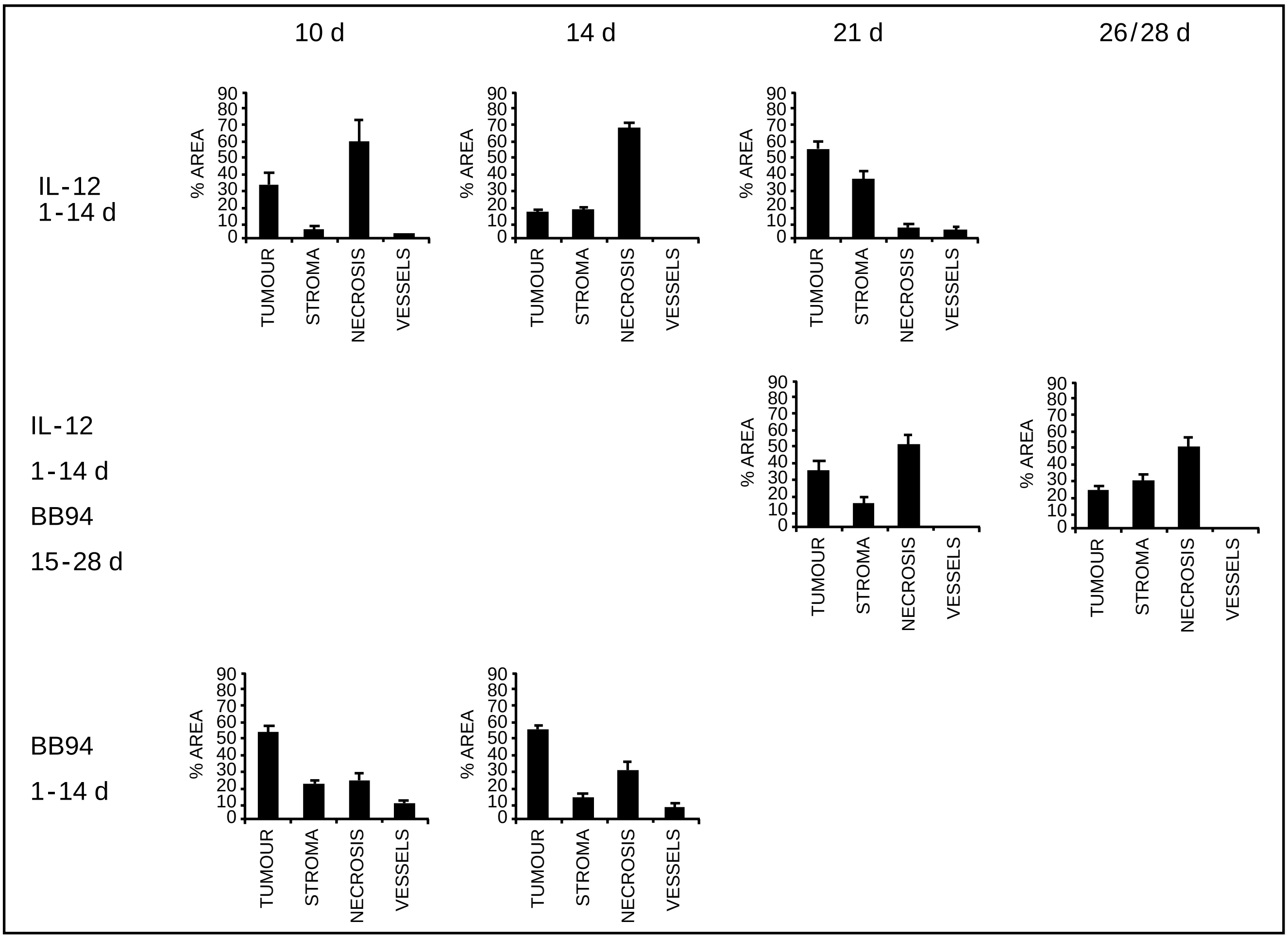

**Figure 4** Analysis of tumour composition following sequential treatments. Percentage areas occupied by tumour cells, stroma, blood vessels and necrosis were measured from haemotoxylin and eosin stained sections as described in materials and methods. Complete sections were analysed from at least 2 mice (2 sections per mouse) from each group at a given time point. This was between 18–50 fields of view. Results were expressed as mean % area (±SEM)

occurs after the cessation of short-term IL-12 therapy. This was confirmed by image analysis and quantitation of the areas occupied by tumour cells, stroma, necrosis or large blood vessels in tumour sections examined at low power. As shown in Figure 4, treatment with BB94 alone for 10 days had little effect on the composition of the tumour compared to placebo mice (data not

© 2000 Cancer Research Campaign 

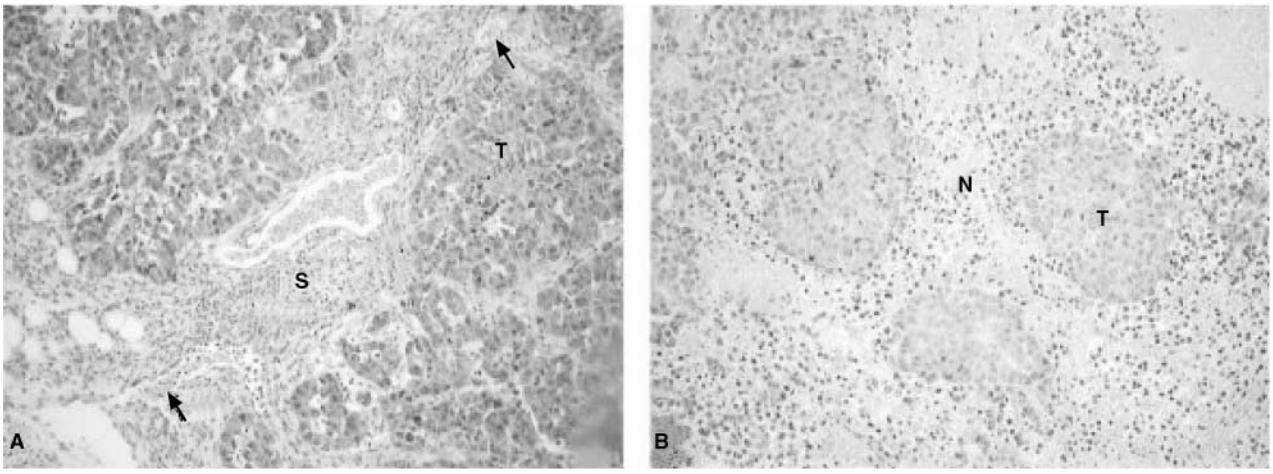

**Figure 5** H&E sections of tumours treated with IL-12 or IL-12/BB94 at 21 days. Tumours treated for 14 days with IL-12 (**A**) show regeneration of blood vessels (arrows) and tumour cell islands (T) surrounded by stroma (S). Sequential treatment with BB94 (**B**) prevented blood vessel regrowth and maintained the high level of necrosis (N) observed at 14 days of IL-12 therapy. Tumour islands were still present following sequential treatment with BB94

shown). The necrotic areas were small and the stromal content was slightly increased compared with IL-12 treated tumours (11.5% versus 5% at 10 days). Tumours from IL-12 treated mice showed signs of regression between 10 and 14 days of therapy. The mean area occupied by epithelial tumour cells decreased from 35% to 15%. By 14 days a mean of 68% of the tumour area was occupied by necrosis and the large blood vessels that characterize this tumour were absent. The effects of IL-12 on the tumour micro-environment were, however, reversible. Seven days after the cessation of IL-12 treatment (day 21), the percentage tumour cell area had increased approximately 4-fold from 15% to 56%; the areas of necrosis were reduced 10-fold from 58% to 6% and new blood vessels had been formed (0% to 4.5% of total area) (Figure 4 and Figure 5A).

When BB94 was administered after IL-12 treatment this regeneration of tumour tissue was not seen either after 21 or 26–28 days; 52% of the tumour was still necrotic and large blood vessels had not re-formed (Figure 4 and Figure 5B).

### DISCUSSION

Systemic IL-12 administration successfully modulates the tumour microenvironment leading to regression and cure in a variety of tumour models (Oshikawa et al, 1999; Seetharam et al, 1999; Silver et al, 1999; Mazzolini et al, 2000) but its use in animals and human patients is limited by toxicity. In the HTH-K breast carcinoma, IL-12 therapy led to tumour regression by increasing IFN-γ production, promoting tumour cytotoxic T-cell infiltrates and producing an anti-angiogenic response (Dias et al, 1998a). The latter involved reduction of VEGF protein, an induction of IP-10 at the tumour site, decreased MMP-9 production and increased local release of its inhibitor TIMP-1 (Dias et al, 1998b).

Since one of the changes induced by IL-12 in the tumour microenvironment was the modulation of MMP-9 and TIMP-1, we suggested that sequential administration of a synthetic MMP inhibitor after IL-12 treatment might produce similar anti-tumour effects with a significant reduction in toxicity.

BB94 is a broad range MMP inhibitor with reported anti-tumour effects in other tumour models (Low et al, 1996; Prontera et al, 1999; Wylie et al, 1999). Its mechanisms of action are not fully understood, but because of its MMP-blocking activities it may change the stromal content of solid tumours and create a solid capsule around the developing tumour (Davies et al, 1993).

Sequential treatment of HTH-K bearing mice with IL-12 followed by BB94 produced anti-tumour effects resulting in an overall survival increase compared with mice treated with IL-12 alone. Tumours treated with this combination had increased tumour necrosis, decreased tumour and stromal areas and were less vascularized than tumours treated with IL-12 alone. Because BB94 alone produced only marginal anti-tumour effects, these results suggest that the combination produced synergistic rather than additive effects. The most significant effect of BB94 administration was to delay tumour regrowth following cessation of IL-12 therapy. Tumours treated with IL-12 alone began to recover 7–10 days after therapy was stopped as characterized by an increase in blood vessels and reduction in necrosis.

Overall, the increase in survival and the anti-tumour effects in the combination group were similar to those seen with prolonged IL-12 therapy alone (Dias et al, 1998a). Prolonged daily IL-12 therapy for more than 14 days of HTH-K induced signs of toxicity. Lethargy, increased coat ‘ruffling’, and in some cases, minor weight loss were observed. Moreover, splenomegaly due to extramedullary haematopoiesis and hepatic necrosis were also observed by histology. In mice treated with 14 day cycles of IL-12 and BB94 the signs of toxicity were less pronounced. Lethargy and coat changes were not seen, no weight loss was observed and hepatic necrosis was reduced. Moreover, in mice treated with IL-12/BB94/IL-12 signs of toxicity were detected after 14 days of IL-12 treatment but were reduced during MMPI administration. Therefore, mice could be treated once more with IL-12 without a corresponding increase in toxicity.

MMPs have been implicated in angiogenesis (Hiraoka et al, 1998; Sang, 1998; Kraling et al, 1999), and because of its MMP blocking effects it has been suggested that BB94 might have potent anti-angiogenic effects. Moreover, in the HTH-K model a decrease in VEGF levels correlated with a reduction in tumour vascularity after prolonged IL-12 treatment (Dias et al, 1998b). Following short-term IL-12 treatment blood vessel regrowth occurred. This was blocked by sequential treatment with BB94

 *© 2000 Cancer Research Campaign*

indicating that at least 1 metalloenzyme (MMP-9) is crucial for angiogenesis in the HTH-K model. Other MMPs or adamalysin metalloproteinases may also be involved. Preliminary data suggests that BB94 also reduced the levels of VEGF within the tumour. We found that tumours which are responsive to BB94 (i.e. no significant increase in tumour volume) contain almost 4-fold lower VEGF levels compared with non-responsive tumours treated with IL-12/BB94 or those treated with IL-12 alone (data not shown).

BB94 may modulate the tumour extracellular matrix composition and upon contact with different ECM components, VEGF production by tumour cells may be reduced. Alternatively, since it has been shown that VEGF binds ECM and can be released by proteolytic cleavage (Park et al, 1993), inhibition of MMP activity by BB94 may lead to the accumulation of VEGF in the ECM making it unavailable for the proliferating tumour endothelium. In the absence of VEGF the tumour endothelial cells undergo apoptosis (Benjamin and Keshet, 1997), halting the angiogenic process and thus limiting tumour growth. This mechanism of action of BB94 has been recently suggested (Bergers et al, 1999). Investigation into the efficacy of BB94 in the RIP1-Tag2 model of pancreatic islet cell carcinogenesis revealed that inhibition of MMP activity reduced the incidence of angiogenic switching in a prevention trial but could only slow tumour growth when administered after tumour establishment had occurred (Bergers et al, 1999). In the HTH-K model, BB94 did not significantly prolong survival alone but could slow tumour recovery following treatment with IL-12.

This, to our knowledge is the first report of combination of an MMP inhibitor with IL-12. We suggest that detailed studies of tumour microenvironment changes may lead to the design of successful biological therapy combinations to target different tumour–stroma interactions.

### ACKNOWLEDGEMENTS

We are grateful to Mike Bradburn (ICRF Medical Statistics Group, Institute for Health Sciences, Oxford, UK) for assistance with statistical evaluation; Stan Wolf (Genetics Institute, Cambridge, Mass) and British Biotech Pharmaceuticals (Oxford, UK) for supplying reagents; Mr G Elia for histological processing of the tumours; and Dr F Burke and Professor I Hart for critically reviewing this manuscript.

### REFERENCES

Benjamin LE and Keshet E (1997) Conditional switching of vascular endothelial growth factor (VEGF) expression in tumors: induction of endothelial cell shedding and regression of hemangioblastoma-like vessels by VEGF withdrawal. *Proc Natl Acad Sci USA* **94**: 8761–8766

Bergers G, Javaherian K, Lo KM, Folkman J and Hanahan D (1999) Effects of angiogenesis inhibitors on multistage carcinogenesis in mice. *Science* **284**: 808–812

Cavallo F, Di Carlo E, Butera M, Verrua R, Colombo MP, Musiani P and Forni G (1999) Immune events associated with the cure of established tumors and spontaneous metastases by local and systemic interleukin 12. *Cancer Res* **59**: 414–421

Davies B, Brown PD, East N, Crimmin MJ and Balkwill FR (1993) A synthetic matrix metalloproteinase inhibitor decreases tumor burden and prolongs survival of mice bearing human ovarian carcinoma xenografts [published erratum appears in Cancer Res 1993 Aug 1;53(15):3652]. *Cancer Res* **53**: 2087–2091

Dias S, Thomas H and Balkwill F (1998a) Multiple molecular and cellular changes associated with tumour stasis and regression during IL-12 therapy of a murine breast cancer model. *Int J Cancer* **75**: 151–157

Dias S, Boyd R and Balkwill F (1998b) IL-12 regulates vegf and mmps in a murine breast-cancer model. *Int J Cancer* **78**: 361–365

Fisher SJ and Werb Z (1995) The catabolism of extracellular matrix components. In: Haralson MA and Hassell JR (eds) *Extracellular Matrix*. The Practical Approach Series. Oxford University Press, Oxford, pp. 261–287

Hiraoka N, Allen E, Apel IJ, Gyetko MR and Weiss SJ (1998) Matrix metalloproteinases regulate neovascularization by acting as pericellular fibrinolysins. *Cell* **95**: 365–377

Kraling BM, Wiederschain DG, Boehm T, Rehn M, Mulliken JB and Moses MA (1999) The role of matrix metalloproteinase activity in the maturation of human capillary endothelial cells in vitro. *J Cell Sci* **112**: 1599–1609

Low JA, Johnson MD, Bone EA and Dickson RB (1996) The matrix metalloproteinase inhibitor batimastat (BB-94) retards human breast cancer solid tumor growth but not ascites formation in nude mice. *Clin Cancer Res* **2**: 1207–1214

Mazzolini G, Qian C, Narvaiza I, Barajas M, Borras-Cuesta F, Xie X, Duarte M, Melero I and Prieto J (2000) Adenoviral gene transfer of interleukin 12 into tumors synergizes with adoptive T cell therapy both at the induction and effector level. *Hum Gene Ther* **11**: 113–125

Myers KJ, Eppihimer MJ, Hall L and Wolitzky B (1998) Interleukin-12-induced adhesion molecule expression in murine liver. *Am J Pathol* **152**: 457–468

Nastala CL, Edington HD, McKinney TG, Tahara H, Nalesnik MA, Brunda MJ, Gately MK, Wolf SF, Schreiber RD, Storkus WJ and et al. (1994) Recombinant IL-12 administration induces tumor regression in association with IFN-gamma production. *J Immunol* **153**: 1697–1706

Ogawa M, Yu WG, Umehara K, Iwasaki M, Wijesuriya R, Tsujimura T, Kubo T, Fujiwara H and Hamaoka T (1998) Multiple roles of interferon-gamma in the mediation of interleukin 12- induced tumor regression. *Cancer Res* **58**: 2426–2432

Oshikawa K, Shi F, Rakhmilevich AL, Sondel PM, Mahvi DM and Yang NS (1999) Synergistic inhibition of tumor growth in a murine mammary adenocarcinoma model by combinational gene therapy using IL-12, pro-IL- 18, and IL-1beta converting enzyme cDNA. *Proc Natl Acad Sci USA* **96**: 13351–13356

Park JE, Keller GA and Ferrara N (1993) The vascular endothelial growth factor (VEGF) isoforms: differential deposition into the subepithelial extracellular matrix and bioactivity of extracellular matrix-bound VEGF. *Mol Biol Cell* **4**: 1317–1326

Prontera C, Mariani B, Rossi C, Poggi A and Rotilio D (1999) Inhibition of gelatinase A (MMP-2) by batimastat and captopril reduces tumor growth and lung metastases in mice bearing Lewis lung carcinoma. *Int J Cancer* **81**: 761–766

Sang QX (1998) Complex role of matrix metalloproteinases in angiogenesis. *Cell Res* **8**: 171–177

Seetharam S, Staba MJ, Schumm LP, Schreiber K, Schreiber H, Kufe DW and Weichselbaum RR (1999) Enhanced eradication of local and distant tumors by genetically produced interleukin-12 and radiation. *Int J Oncol* **15**: 769–773

Silver DF, Hempling RE, Piver MS and Repasky EA (1999) Effects of IL-12 on human ovarian tumors engrafted into SCID mice. *Gynecol Oncol* **72**: 154–160

Tannenbaum CS, Wicker N, Armstrong D, Tubbs R, Finke J, Bukowski RM and Hamilton TA (1996) Cytokine and chemokine expression in tumors of mice receiving systemic therapy with IL-12. *Journal of Immunology* **156**: 693–699

Tare NS, Bowen S, Warrier RR, Carvajal DM, Benjamin WR, Riley JH, Anderson TD and Gately MK (1995) Administration of recombinant interleukin-12 to mice suppresses hematopoiesis in the bone-marrow but enhances hematopoiesis in the spleen. *J Interferon Cytokine Res* **15**: 377–383

Tsung K, Meko JB, Peplinski GR, Tsung YL and Norton JA (1997) IL-12 induces T helper 1-directed antitumor response. *Journal of Immunology* **158**: 3359–3365

Wylie S, MacDonald IC, Varghese HJ, Schmidt EE, Morris VL, Groom AC and Chambers AF (1999) The matrix metalloproteinase inhibitor batimastat inhibits angiogenesis in liver metastases of B16F1 melanoma cells. *Clin Exp Metastasis* **17**: 111–117

© 2000 Cancer Research Campaign
